# The Somatic Engram and Reversal of Allostatic Load via Reconsolidation: A Technical Description of the RB7™ Protocol

**DOI:** 10.7759/cureus.101672

**Published:** 2026-01-16

**Authors:** Rafael M Barreiros

**Affiliations:** 1 Neurosciences, Instituto Rafael Barreiros, Londrina, BRA

**Keywords:** allostatic load, false memories, implicit memory, memory reconsolidation, prediction error, psychosomatic medicine, somatic engram, trauma treatment

## Abstract

Developmental neurobiology indicates that baseline calibration of the hypothalamic-pituitary-adrenal (HPA) axis occurs predominantly during critical periods of plasticity (zero to five years). Early trauma may generate lifelong Allostatic Load, manifesting as a probable physiological substrate of psychosomatic disorders, treatment-resistant anxiety, and systemic dysfunctions, including chronic pain and metabolic sensitivities. Conventional therapies often operate via extinction mechanisms, which may suppress rather than modify the original fear trace.

This article describes the RB7™ Protocol (Reconsolidation & Biology 7-Steps), a structured intervention designed to induce Memory Updating through interoceptive Mismatch Prediction Error. The methodology prioritizes biological validity over historical veracity (focusing on the functional engram regardless of factual accuracy). The protocol consists of seven sequential steps: (1) Hypothesis Mapping and Safety Calibration; (2) Vagal Modulation; (3) Engram Reactivation; (4) Heuristic Tracking using somatic bridging to bypass System 2; (5) Updating via Radical Reality; (6) Immediate Verification; and (7) Real-World Verification. This sequence aims to replicate the neurochemical conditions required for memory labilization.

By utilizing somatic evidence of survival to overcome extinction-based barriers, the protocol presents a theoretically coherent mechanism for rewriting traumatic engrams. While current findings are based on preliminary multiple-case series, they suggest Affective Neutrality as a clinical marker of allostatic reversal. However, future randomized controlled trials (RCTs) are required to establish statistical generalizability.

## Introduction

Developmental foundations of Allostatic Load

Contemporary developmental neurobiology indicates that the baseline calibration of the hypothalamic-pituitary-adrenal (HPA) axis occurs predominantly during critical periods of plasticity, particularly within the first five years of life [1]. Early exposure to adverse childhood experiences (ACEs) generates distinct morphometric alterations in threat-processing regions, rendering the amygdala hypersensitive to potential danger. This early recalibration functions as an adaptive survival mechanism in a hostile environment, but manifests later as Allostatic Load - the cumulative physiological wear and tear resulting from chronic neuroendocrine dysregulation. The persistence of refractory pathologies - from fibromyalgia to generalized anxiety disorders - suggests that this clinical dysregulation is not merely cognitive, but sustained by this autonomous neural architecture.

The Vertical Perspective and Vulnerability Windows

Recent human studies expand this perspective. Teicher and Samson [2] demonstrate that specific developmental windows render distinct brain regions - such as the hippocampus or prefrontal cortex - selectively vulnerable to particular trauma types. These data support the need for a precise developmental chronology when tracing the root engram, aligning with the Vertical Perspective of stress adaptation, where current symptoms are expressions of past biological survival strategies.

The Somatic Engram

Within this context, the therapeutic target is defined as the Somatic Engram. Structurally, this engram resides in implicit procedural memory; functionally, it operates as a stabilized interoceptive prediction. It is not a passive archive of the past, but an internal action model that perpetually simulates a physiological threat state. Crucially, adult life experiences rarely modify this basal schema spontaneously. Rather, they tend to operate through a mechanism of neurobiological confirmation bias, whereby the brain selectively filters the environment to locate evidence that validates the original danger prediction. Thus, each new stressful event may function as structural reinforcement through Long-Term Potentiation (LTP), consolidating the neural architecture of early trauma [3].

Clinical imperative and limitations of standard care

The clinical imperative for such a protocol is underscored by the limitations of current standards of care. Epidemiological data suggest that approximately 30-50% of patients with anxiety and stress-related disorders fail to achieve full remission with conventional cognitive-behavioral or pharmacological interventions [4,5]. This high rate of treatment resistance contributes significantly to the global burden of disease, highlighting an urgent need for mechanism-based interventions that target the root biological drivers of allostatic load rather than symptom management alone.

Mechanism-based resolution

While conventional therapy frequently seeks to inhibit expression of this circuit through Extinction, the biological imperative for stable resolution requires protein-level destabilization of these procedural connections - a process that the literature suggests can be induced by violating the original prediction (Mismatch Prediction Error). While modalities such as EMDR (Eye Movement Desensitization and Reprocessing) and Coherence Therapy have successfully leveraged aspects of these principles, the RB7 Protocol seeks to operationalize these mechanisms into a reproducible, seven-step algorithm. Specifically, it distinguishes itself by emphasizing the preliminary reversal of autonomic dysregulation (via vagal modulation) as a biological prerequisite for accessing the labile engram.

Objective

Based on this neurobiological rationale, the objective of this technical report is to describe a structured clinical framework - the RB7™ Protocol - designed to induce mismatch-mediated reconsolidation for the reversal of traumatic Allostatic Load. This report details the operational steps required to locate, reactivate, and update implicit survival predictions, offering a standardized methodology for clinical application and future efficacy research.

## Technical report

**Figure 1:**
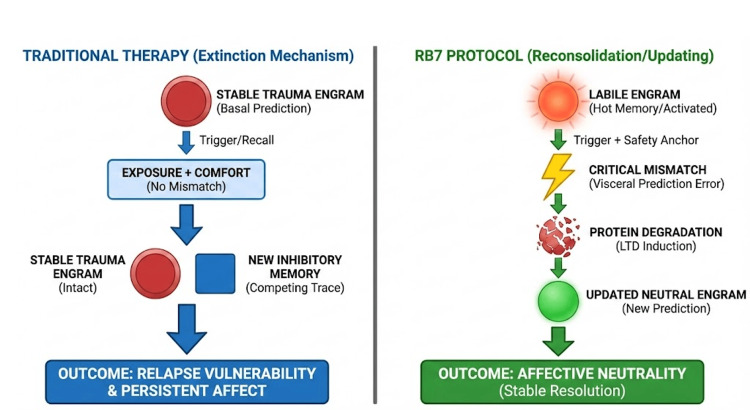
Schematic comparison of neurobiological treatment mechanisms Left (traditional therapy): extinction mechanism generates a new inhibitory memory (blue square) competing with the intact original traumatic engram (red circle). Right (RB7 Protocol): reconsolidation mechanism uses Prediction Error (Mismatch) to induce labilization and protein degradation (LTD) of the original trace, resulting in its replacement by an updated neutral engram (green circle)

The method is structured into seven sequential steps designed to meet the stringent neurochemical conditions for memory labilization (opening) and reconsolidation (rewriting) [6], integrating contemporary understanding of memory malleability and cognitive systems (Figure 1).

Phase I: access, safety, and labilization

Step 1: Hypothesis Mapping and Neuroceptive Safety Calibration

Unlike static diagnostic models, the RB7™ Protocol recognizes that access to the precise root of trauma (the Somatic Engram) is often inaccessible via direct cognitive narrative. Therefore, the first stage aims to establish working hypotheses and build the safety architecture required for neural navigation.

1.1. Anamnesis as a heuristic compass: The limbic system does not primarily process verbal logic. The initial report is treated as the cognitive "tip of the iceberg." The functional root of the symptom resides in implicit associative networks. Step 1 functions as heuristic tracking:

Reference identification: Memories, sensations, and family patterns serve as entry triggers.

Route establishment: Hypotheses are generated regarding how the symptom (e.g., inflammation) connects to life history (e.g., parentification).

Methodological uncertainty: It is assumed that the initial memory rarely sustains the symptom. The method uses emotion as the guiding thread (Somatic Bridge); anamnesis defines direction, but physiology determines the path.

1.2. The therapeutic bond as a modulator of lability: Memory updating requires a paradoxical state: high emotional arousal within a safety window. Based on Schwabe et al. [7], the following is proposed: Stress without safety tends to induce defensive responding, limiting hippocampal access. Stress with safety facilitates plasticity. The therapist functions as a temporary secure attachment figure, enabling access to aversive memories without dissociation.

1.3. Clinical procedure (feedback): The therapist presents a Provisional Coherence Map, validating the protective function of the symptom to reduce limbic resistance and engage patient collaboration.

Step 2: Vagal Modulation and Affective Safety Priming

The protocol diverges from deep relaxation approaches, aiming instead for alert safety.

2.1. Neurobiological rationale: Labilization requires moderate noradrenergic activation in the amygdala, avoiding states of dorsal vagal shutdown [7]. Excessive parasympathetic relaxation may reduce catecholaminergic signaling below the threshold necessary for protein destabilization [8]. The protocol targets the "window of tolerance," in which the patient experiences intense emotion while remaining anchored in regulated physiology.

2.2. The physiological maneuver: The "physiological sigh" is used to support ventral vagal regulation, instructing the patient to prioritize bottom-up (bodily sensation) processing over top-down cognition.

2.3. The emotional catalyst: A Safety Script is read to validate pain and provide safety simultaneously (e.g., "It makes sense that your body is exhausted... but today you are safe here with me."). The objective is to facilitate the emergence of Hot Memory within a controlled environment.

Step 3: Engram Reactivation via Visceral Anchoring and Field Perspective

The patient evokes the somatic sensation using a Referential Memory. This step establishes the Somatic Benchmark (baseline).

3.1. Field perspective: The protocol requires first-person re-experiencing (seeing through one's own eyes), avoiding observer dissociation. This aims to recruit sensorimotor regions associated with the original event.

3.2. Retrieval mechanism: The literature [6,9] suggests that plasticity depends on circuit activation.

Somatic signature: Interoceptive salience (e.g., tachycardia, tension) in the "here and now."

Function: This physiology guides tracking in Step 4.

3.3. Verification contract: Discomfort intensity in this memory serves as a comparative measure for Step 7.

Phase II: synaptic updating and biological reality

Step 4: Heuristic Tracking and Access to Subjective Reality

The objective of this phase is to identify the specific autobiographical memory or implicit schema that fuels the current symptom. The protocol posits that every dysregulated emotional reaction is a valid response to a past learning event that has been generalized. Therefore, the clinician guides the patient to trace the "affective bridge" from the present symptom back to the original sensitizing event (the root engram).

4.1. Judgment bypass and Affect Bridge: Operationally, this is achieved through the technique of Somatic Bridging (adapted from the "Affect Bridge" technique; [10]) combined with temporal urgency. The clinician directs the patient’s attention to the visceral sensation of the current symptom (e.g., "tightness in the chest") and utilizes rapid-fire questioning to request the immediate surfacing of associated images or phrases. By enforcing a response latency of less than 3 seconds, the protocol intentionally overloads the explicit processing capacity of the Prefrontal Cortex (System 2) [11]. This prevents cognitive rationalization and forces the brain to retrieve information directly from implicit procedural memory (System 1) [12], often revealing the root engram in its raw, emotional form rather than as a polished narrative. Once the root engram is identified and the patient displays signs of emotional resonance (affective activation), the target is considered "locked" and available for the updating phase.

4.2. Memory as a plastic construction: Evidence that memory is malleable is accepted [13]. The protocol does not pursue "forensic truth," but functional truth. It is understood that constructed narratives can become integrated into autobiographical memory. Therefore, whatever imagery surfaces under the high-speed induction of Section 4.1 is treated as the valid operational target.

4.3. Biological versus historical validity: Studies indicate that false beliefs can produce real physiological responses [14]. Consequently, the therapeutic target is the biologically sustained reaction supported by memory (the functional engram), not necessarily the event's historical precision. If the retrieved memory generates somatic activation, it is biologically valid for reconsolidation, regardless of its factual accuracy.

Step 5: Updating through Radical Reality and Visceral Mismatch Prediction Error

This step constitutes the inflection point. With the memory activated, the patient is guided to confront biological evidence.

5.1. Intertemporal juxtaposition: The "adult self" (survivor) is positioned before the "child self" (danger prediction). Instruction: "Tell her the reality. Tell her that you survived."

5.2. Strategic exclusion of compensatory comfort (Anti-Reparenting): Unlike approaches that seek to comfort the inner child (Reparenting), RB7™ avoids palliative consolation based on the hypothesis that it may primarily facilitate Extinction learning rather than Reconsolidation.

Neurobiological Rationale

Current literature distinguishes two dominant outcomes of memory retrieval: Extinction (involving prefrontal inhibition of the amygdala) and Reconsolidation (involving protein-synthesis dependent updating). As demonstrated by Sevenster et al. [15] and Pedreira et al. [16], the transition to reconsolidation is strictly governed by a specific Prediction Error. The protocol posits that providing imagined safety ("You are safe now") creates a competing safety memory (Extinction) but leaves the original threat prediction intact. In contrast, RB7™ targets the falsification of the threat itself. By juxtaposing the "child's expectation of annihilation" with the "adult's biological survival," the protocol induces the necessary Mismatch Prediction Error (surprise). This discrepancy aims to destabilize α-amino-3-hydroxy-5-methyl-4-isoxazolepropionic acid (AMPA) receptors and overwrite the "helplessness" rule with "resilience," rather than merely inhibiting it.

Risk Management and Dissociation

While the protocol demands high emotional activation to ensure such destabilization, the clinician must actively monitor for signs of peri-traumatic dissociation (e.g., freezing, blank staring, or verbal unresponsiveness). Consistent with the inverted-U function of stress on memory described by Schwabe et al. [8], excessive hyperarousal beyond the window of tolerance may hinder reconsolidation. Therefore, if the patient displays signs of shutdown, the "radical reality" confrontation is temporarily paused. The clinician briefly re-engages the vagal modulation techniques from Step 2 (specifically the physiological sigh) solely to restore cortical processing capacity, immediately returning to the mismatch procedure once the patient is stabilized.

Phase III: consolidation and verification (the stress test)

Step 6: Immediate Verification

The verification of reconsolidation requires confirming that the original trace has been silenced, not merely suppressed.

Procedure: The clinician guides the patient to vividly visualize the same traumatic reference cues that previously elicited distress (Step 3). The patient is instructed to scan their body for any residual activation while holding the image of the event details.

Criteria for success: A successful intervention is marked by a "Biological Null Response"-the complete absence of autonomic arousal (e.g., tight chest, rapid heartbeat) despite clear cognitive recall of the event. The patient typically reports that the memory feels "distant" or "neutral."

Biomarker correlation: While clinical assessment relies on somatic reporting, this protocol acknowledges that objective biomarkers, such as Heart Rate Variability (HRV), can serve as supplementary confirmation of this shift from sympathetic dominance to vagal regulation, as noted by the reviewer’s suggestion for robust validation in research settings.

6.2. LTP of the new prediction: Re-experiencing the prior memory without autonomic activation reinforces the new neural pathway. Through immediate repetition, the brain learns that the visual stimulus no longer recruits the amygdala.

Step 7: Real-World Verification Through Collapse of the Referential Memory Return to the Referential Memory From Step 3

7.1. Associative Collapse (Fractal Cleaning): The protocol hypothesizes that symptoms are not isolated but organized in a dependency hierarchy around the root engram.

Clinical observation: This phenomenon was consistently documented in the preliminary multiple case series underpinning this report. Upon successful updating of the core survival prediction (Step 5), patients reported spontaneous remission of secondary and tertiary triggers that were not directly targeted during the session. This suggests that once the foundational "threat node" is deactivated, the dependent neural network - deprived of its sustaining emotional charge - undergoes a structural collapse, validating the efficiency of targeting the root rather than the branches.

7.2. Reality feedback (Biological Null-Response): The final test is autonomic rather than subjective. The patient actively attempts to trigger the recent memory. Success is declared when the body remains in a state of ventral vagal relaxation, indicating deactivation of the prediction error generator.

Clinical Efficiency and Protocol Duration

The protocol structure consists of one foundational Hypothesis Mapping session followed by a targeted series of Memory Updating sessions. The duration of treatment is not arbitrary but dictated by the density of the prediction network identified during mapping. While focused dysregulations (e.g., specific phobias, burnout, chronic pain clusters) are typically resolved within 5 regression sessions, complex systemic adaptations (such as metabolic resistance/obesity or Generalized Anxiety Disorder) may require up to 12 sessions. This variability exists because a single pathology is rarely sustained by one engram, but by a multifaceted architecture of survival predictions (e.g., social anxiety may rely on distinct predictions of judgment, abandonment, and betrayal). Therefore, the protocol concludes only when all mapped predictions driving the allostatic load have been individually updated, ensuring a complete collapse of the symptomatic structure.

## Discussion

Mechanistic differentiation: evolution relative to traditional therapeutics

The RB7™ Protocol is not proposed merely as a variation of existing therapies, but as an applied framework of memory reverse engineering. Its distinction is grounded in five neurobiological pillars that address structural limitations of prior models.

Noradrenergic Optimization vs. Trance State

Whereas classical hypnotherapy frequently targets deep trance states (characterized by theta/delta activity), RB7™ maintains the patient strictly within the "window of tolerance." Memory labilization requires moderate noradrenergic activation. Schwabe et al. [8] indicate that hypoactivation (excessive relaxation) fails to labilize the memory trace, while hyperactivation promotes dissociation and blocks reconsolidation. RB7™ aims for the optimal arousal range required for synaptic plasticity, avoiding the sedation often found in relaxation-based therapies.

Neutrality vs. Morality (Elimination of Catharsis and Forgiveness)

The protocol explicitly rejects abreaction (e.g., shouting or hitting pillows), as motor execution of defense without a change in prediction may inadvertently reconsolidate the fear pathway rather than alter it. Likewise, "forgiveness therapy" is excluded from the protocol. Forgiveness is a cognitive moral construct and is ineffective at the level of the amygdala. Because the protocol relies on irrefutable somatic evidence of survival, the aggressor becomes biologically irrelevant to the patient's safety. The clinical target is Affective Neutrality, not moral absolution. Crucially, this distinction is applied specifically to the target of implicit procedural memory. While cognitive moral structures (System 2) remain essential for social navigation and ethical decision-making, the protocol posits that they are mechanistically insufficient to overwrite the subcortical prediction errors driving somatic dysregulation. Therefore, the therapeutic bypassing of moral logic is not a rejection of cognitive values, but a strategic necessity to access the non-verbal, survival-based encoding of the traumatic engram.

Survival Evidence vs. Reparenting (Transformation vs. Competition)

RB7™ replaces the "inner-child comfort" model (reparenting) with "survival proof." Reparenting techniques may generate a competing memory (comfort) that attempts to inhibit the pain, but does not erase the original threat prediction. RB7™ uses the biological fact of survival to nullify the death prediction. The patient is not comforted in their pain; the patient is validated in their evolutionary victory. This is proposed to induce structural updating (Long-Term Depression [LTD] of the fear pathway) rather than competitive inhibition.

Activation via Referential Memory vs. Hypnotic Suggestion

Unlike hypnotherapy, which often relies on abstract suggestions, RB7™ strictly uses recent Referential Memories to activate physiology. This ensures the nervous system is responding to a real, active engram (a "Hot Memory"), rather than a transient imaginative construction. This increases the likelihood that the intervention reaches the neurochemical root.

Validation of Helplessness vs. Positive Affirmation (Radical Reality)

The protocol prohibits compensatory positive affirmations (e.g., “you are strong”) during the phase of trauma activation. Informing the limbic system that it is strong while it is re-experiencing the terror of helplessness produces a negative Prediction Error (a biological falsehood), which can induce resistance. RB7™ adopts "Radical Reality": the phenomenological helplessness of the child is validated (“You were small and could not defend yourself”). By accepting the truth of past helplessness and juxtaposing it with the irrefutable reality of present survival, the brain resolves the paradox without cognitive dissonance.

Limitations and future directions

It is imperative to acknowledge the limitations inherent in the current level of evidence. The efficacy data supporting the RB7™ protocol are currently derived from the protocol developer's preliminary multiple case series and observational clinical practice. Notably, these studies suggest applicability in anxiety disorders, chronic pain, and metabolic dysfunctions [17,18,19]. However, to maintain scientific integrity, it is explicitly disclosed that these findings are derived from the developer’s clinical series, which carries an inherent risk of observer and selection bias. Furthermore, while the protocol targets the reconsolidation window, clinicians must be cautious regarding patient hyperactivation; if stress levels exceed the therapeutic window, it may lead to dissociation rather than updating, requiring immediate vagal stabilization. Additionally, future investigations should incorporate objective biomarkers, such as Heart Rate Variability (HRV), to complement subjective self-reports of "Affective Neutrality."

Consequently, the protocol has not yet been subjected to large-scale, double-blind randomized controlled trials (RCTs). Until such independent validation is conducted, the clinical superiority of the RB7™ framework over standard-of-care interventions (e.g., CBT or EMDR) remains a hypothesis. This report serves to define the standardized framework necessary for such future rigorous experimental investigations. Memory Ethics Consistent with Loftus [20], the accessed memories are treated as the patient's "psychic reality" and should not be used as factual evidence in legal contexts without external corroboration. The protocol operates on functional biological truth rather than forensic precision.

## Conclusions

The RB7™ Protocol presents a structured and biologically coherent methodology for the resolution of traumatic engrams. By prioritizing memory reconsolidation mechanisms - specifically the generation of Mismatch Prediction Error - over emotional catharsis or cognitive coping, it addresses the physiological root of Allostatic Load. This approach represents a potential paradigm shift from symptom management to the stable physiological resolution of traumatic memory, suggesting that lasting clinical change requires satisfying specific neurochemical conditions rather than merely altering cognitive narratives. The protocol offers a replicable framework for clinicians seeking to intervene at the level of implicit procedural memory. However, consistent with the current hierarchy of evidence, broad clinical extrapolation should be accompanied by further validation through large-scale RCTs to confirm the statistical generalizability of these preliminary findings.
